# Quality of life and burden during the COVID-19 pandemic: a longitudinal study with family caregivers of persons living with Alzheimer’s disease

**DOI:** 10.3389/fpsyg.2026.1662182

**Published:** 2026-05-20

**Authors:** Laura Brito, Susana Faria, Angela Leite, M. Graça Pereira

**Affiliations:** 1 School of Psychology, University of Minho, Braga, Portugal; 2 Mathematics Department, University of Minho, Braga, Portugal; 3 Center for Philosophical and Humanistic Studies, Catholic University, Braga, Portugal

**Keywords:** Alzheimer’s disease (AD), burden, caregiving, COVID-19, forgiveness, longitudinal, quality of life

## Abstract

**Introduction:**

The COVID-19 pandemic intensified the challenges faced by family caregivers of persons living with Alzheimer’s disease, potentially affecting their psychological, behavioral, and physiological well-being. Understanding how these processes evolve over time is critical for informing targeted interventions.

**Methods:**

A longitudinal study was conducted with 130 Portuguese family caregivers assessed at three time points over a 12-month period. Measures included quality of life (QoL), caregiver burden, psychological distress, forgiveness, health behaviors, caregiving competence, family stress, and heart rate variability (HRV). Linear mixed-effects models and mediation analyses were performed while controlling for caregiver age and dementia severity.

**Results:**

Physical QoL and health behaviors initially declined but subsequently demonstrated a trend toward recovery over time, whereas mental QoL showed a sustained decline. HRV, caregiving competence, and family stress increased throughout the study period. Forgiveness mediated the relationship between psychological distress and both caregiver burden and physical QoL, but not mental QoL. Health behaviors did not mediate the association between HRV and caregiver burden.

**Discussion:**

These findings highlight the dynamic and multifaceted nature of caregiving under prolonged stress conditions. Although caregivers demonstrated adaptive processes over time, the persistent decline in mental QoL underscores their continued psychological vulnerability. The mediating role of forgiveness suggests that it may serve as an important protective mechanism and a promising target for interventions aimed at improving caregiver well-being.

## 1 Introduction

Dementia is a growing concern within neurocognitive disorders, profoundly impacting the health and well-being of the elderly and preventing a graceful and dignified aging process. Presently, over 25 million people worldwide grapple with dementia, and it is anticipated that these figures will double within the next two decades. Annually, there are well over 10 million new cases of dementia reported worldwide, with an average life expectancy of 4.5 years post-diagnosis (Alzheimer’s Association, 2021). This condition, previously referred to as dementia, causes impairments in daily functioning, requiring a caregiver’s assistance, particularly for the 10% of individuals over 65 years old and for the 50% over 90 years old who receive a dementia diagnosis (Alzheimer’s Association, 2021).

Alzheimer’s disease (AD) presents various symptoms such as cognitive decline, mood shifts, hostility, communication breakdowns, and hallucinations (Llibre-Rodriguez et al., 2022). Family caregivers (FCs) play diverse roles as advocates and care coordinators, managing appointments, healthcare connections, and medication regimens (Contreras et al., 2021; Reckrey et al., 2020). As dementia advances, persons living with AD increasingly depend on FCs for fundamental tasks like walking, eating, and bathing, often requiring constant FCs’ supervision (Connors et al., 2019). This heightened demand often leads to a decline in the FCs’ quality of life (QoL) and reported personal burden as the disease progresses (Reckrey et al., 2020).

Several classic stress–health process models have been developed to explain the impact of family caregiving on caregivers’ health and well-being. Among them, the Stress Process Model proposed by Pearlin et al. (1990) remains one of the most influential, frameworks, as it emphasizes the role of personal resources in mediating psychological outcomes, a core objective of the present study. This model conceptualizes caregiving as a dynamic and multidimensional process. In the present study, primary stressors were operationalized as disease severity, while secondary stressors comprised role strains (family stress) and intrapsychic strains (psychological distress, personal gains, caregiving competence, and HRV). Mediating conditions, specifically personal resources, included forgiveness and health behaviors, whereas caregiver burden and quality of life were assessed as health-related outcomes. Although burden was initially conceptualized as a primary stressor in the original model, more recent empirical work has operationalized subjective burden as an outcome reflecting caregivers’ cognitive appraisal of stress and perceived strain (Roland and Chappell, 2019). Consistent with this approach, the present study conceptualized subjective burden and quality of life as outcome variables representing psychological and health-related consequences of the caregiving stress process.

The COVID-19 pandemic has deleteriously impacted the already difficult journey of dementia patients. In Portugal, the government declared a state of emergency in March 2020 to curb the spread of the virus (Diário da Republica. Decree No. 2-A/2020 of the Presidency of the Council of Ministers, 2020), implementing measures like physical distancing (Gilbert et al., 2020). Frail individuals, including older adults with chronic conditions, were advised to stay home, placing the full-time care burden on family members who often lacked the requisite skills (Brown et al., 2020). COVID-19 home confinement disrupted routines, leading to increased anxiety, boredom, conflicts with FCs, disorganization, cognitive decline, sleep disturbances, and stress (Korczyn, 2020). Vulnerable elderly individuals faced additional risks due to new rules such as wearing masks and social isolation, especially those with comorbidities (Cohen et al., 2021).

Research on caregiving during COVID-19 showed varied outcomes. Some caregivers experienced severe adverse effects, while others reported positive outcomes despite obstacles (Lightfoot et al., 2021). Conversely, some studies found no increase in burden or decline in QoL (Mitchell et al., 2023). Stressors include infection worries and negative end-of-life concerns (Mitchell et al., 2021), with high levels of anxiety (28.6%) and depression (38.8%) reported in some studies (Lane et al., 2022). FCs of persons living with AD, particularly during the pandemic, reported higher levels of stress and burden compared to FCs of persons living with other conditions due to social isolation. Studies indicated a negative impact on caregivers’ QoL and reduced engagement in health behaviors, with increased stress-related symptoms like sleep issues and difficulty concentrating (Cohen et al., 2021; Savla et al., 2021). The pandemic led to infected or ill FCs, resulting in isolation, mental health issues, and financial constraints. When combined with irregular external care support, such factors increased caregiving burden and reduced QoL (Brown et al., 2020). FCs of persons living with AD experienced anxiety, mood disturbances, sleep issues, unhealthy habits, and decreased support (Carcavilla et al., 2021). These findings suggest that the COVID-19 pandemic may have intensified existing caregiving stress processes. In the present study, COVID-19 is, therefore conceptualized as a macro-level contextual stressor affecting all participants during the period of data collection, rather than as an individual-level predictor. As such, it represents a shared environmental condition that may have amplified psychological, behavioral, and physiological stress responses over time.

The emotionally demanding nature of caring for a family member living with dementia poses significant risks to the well-being of both the caregiver and their family, potentially leading to a decline in QoL (Watson, 2019). Caregiving burden has been associated with psychological distress, including anxiety and depressive symptoms (Allen et al., 2020; Watson, 2019), and these stressors may have been intensified by fear of contagion, social isolation, and reduced access to formal care services (Bailey et al., 2022). Sociodemographic and contextual factors, including female sex, spousal relationship, limited social support, lower caregiving confidence, and greater dementia severity were identified as predictors of increased distress and burden (Wulff et al., 2020; del-Pino-Casado et al., 2021; Wister et al., 2022).

Family stress represents an additional contextual strain within the caregiving system. The irregularity or lack of outside care assistance increases family stress, adding strain to already high caregiver burden (Brown et al., 2020). Cessation of face-to-face activities like support groups further hampers caregivers’ coping abilities. Suspension of formal assistance and care center closures increases caregiver responsibilities, impacting their ability to provide adequate care (Canevelli et al., 2020). Innumerable hours of caregiving may lead to loneliness and intensified stress (Greenberg et al., 2020). Despite challenges, caregiving experiences can also have positive aspects.

FCs frequently report caregiving competence and personal gains in their role (Jeste et al., 2021). Higher caregiving competence and recognition of personal growth were associated with lower subjective burden, greater optimism, and improved psychological well-being (Fekete et al., 2017; García-Castro et al., 2022; Nemcikova et al., 2023; Jeste et al., 2021). These positive dimensions may function as protective resources within the stress process.

Forgiveness is another potential intrapersonal coping resource. Conceptualized as a dispositional tendency to forgive oneself, others, and difficult situations, forgiveness has been associated with lower marital distress, reduced anxiety and depressive symptoms, and lower caregiver burden (DeCaporale-Ryan et al., 2016; Rasmussen et al., 2019). Empirical evidence indicates that forgiveness is associated with improved mental QoL (Kaleta and Mróz, 2020) and may mediate relationships between negative affective states and well-being (Çolak and Güngör, 2021). In dementia caregiving contexts, relational strain and exposure to behavioral symptoms may render forgiveness particularly relevant as a coping mechanism (McGee et al., 2021).

Healthy behaviors such as regular exercise, proper nutrition, and stress management were linked to lower distress and burden and higher QoL (Xue et al., 2021). Conversely, caregiver burden and chronic stress may disrupt self-care routines, contributing to unhealthy behaviors such as sedentary lifestyles, poor sleep, and excessive alcohol use (Hoffman and Zucker, 2016). Sleep disturbances were associated with mood disorders and reduced QoL (Yuan et al., 2020). These findings suggest a bidirectional relationship between caregiving stress and health behaviors, in which stress may impair self-regulation, and health behaviors may influence psychological well-being.

In addition to behavioral processes, caregiving stress is associated with physiological dysregulation. Heart rate variability (HRV), an indicator of autonomic nervous system flexibility, reflects the body’s capacity to adapt to environmental demands. Lower HRV has been linked to chronic stress exposure, cardiovascular risk, and psychological distress (Shaffer and Ginsberg, 2017; Kim et al., 2018). In caregiving populations, higher burden and anxiety have been associated with lower HRV (James et al., 2021). Modifiable lifestyle factors, such as physical activity, diet, and hypertension, are also associated with HRV indices (Speer et al., 2020), highlighting the interconnected nature of behavioral and physiological stress processes. While predominant literature conceptualizes HRV as an outcome of stress, autonomic flexibility has also been associated with self-regulatory capacity, suggesting that physiological regulation may relate to engagement in adaptive behaviors. Given the limited longitudinal evidence in dementia caregiving contexts, the potential role of health behaviors in linking HRV and perceived burden warrants exploratory examination. Within this framework, psychological distress may influence caregivers’ appraisal of burden and quality of life, potentially through intrapersonal coping resources such as dispositional forgiveness. This pathway is consistent with stress appraisal models, suggesting that cognitive-emotional regulation processes may shape how caregiving demands are interpreted and internalized.

In contrast, the interplay between physiological regulation (indexed by HRV), health behaviors, and perceived burden remains less clearly established in Alzheimer’s dementia caregiving research. Given that HRV reflects autonomic flexibility and self-regulatory capacity, it is theoretically plausible that physiological regulation may relate to engagement in adaptive health behaviors, which could in turn be associated with perceived burden. However, empirical evidence, particularly longitudinal, examining these pathways remains limited, warranting exploratory investigation.

Despite growing cross-sectional evidence, longitudinal research simultaneously examining psychosocial, behavioral, and physiological processes within a unified stress framework among dementia FCs remains limited, particularly in the context of the COVID-19 pandemic. Together, these proposed pathways integrate psychological, behavioral, and physiological domains within an extended stress process framework. While the distress–forgiveness pathway represents a primarily cognitive-emotional mediation process, the HRV–health behaviors pathway reflects a hypothesized psychophysiological–behavioral linkage. Examining both pathways longitudinally enables the study of regulatory mechanisms that may contribute to caregiving outcomes beyond direct stress exposure.

Based on the theoretical framework of Pearlin et al. (1990) and Roland and Chappell (2019), this study’s aims and hypotheses over a 12-month period during the COVID-19 pandemic were:

1 To assess changes in QoL and burden over time, controlling for clinical assessment of Alzheimer’s dementia and carer’s age.

*H1*: Over the 12-month study period, FCs will exhibit a significant decrease in QoL (physical and mental) and a significant increase in perceived burden.

2 To assess changes in forgiveness, distress, HRV, caregiving competence, personal gains, health behaviors, and family stress over time, controlling for the clinical assessment of Alzheimer’s dementia and the family caregiver’s age.

*H2*: Across the 12-month period, FCs will show significant increases in psychological distress and family stress, along with worsening behavioral and physiological regulation (decreased engagement in positive health behaviors and reduced HRV). In contrast, intrapersonal coping resources like forgiveness, caregiving competence, and personal gains will remain stable or increase over time.

3 To assess the mediator role of forgiveness between distress and burden/QoL over time.

*H3*: The association between psychological distress and caregiving outcomes (burden, physical QoL, and mental QoL) will be indirectly explained by dispositional forgiveness, such that higher distress will be associated with lower forgiveness, which in turn will be associated with higher burden and poorer quality of life.

4 To assess the mediator role of health behaviors between HRV and burden over time.

*H4*: The association between physiological stress (indexed by HRV) and perceived burden will be indirectly explained by health behaviors, such that lower HRV will be associated with poorer health behaviors, which in turn will be associated with higher burden.

## 2 Materials and methods

### 2.1 Participants

This prospective longitudinal study was conducted between 2021 and 2022. Data were collected in participants’ homes over a 12-month period, coinciding with Portugal’s COVID-19 partial lockdown. Three assessment moments were conducted: baseline (T1), six months later (T2), and twelve months after baseline (T3).

Participants were Portuguese FCs recruited from the IADem Plan in Northern Portugal, which provides research and support services for caregivers and persons living with AD and fragility, including home visits. Inclusion criteria were: (a) being a family caregiver of a relative diagnosed with AD; (b) using the Portuguese National Health Care System; and (cc) being over 18 years of age. Exclusion criteria included being a formal caregiver, currently receiving psychological or psychiatric treatment, or presenting cognitive impairment (assessed using the Mini-Mental State Examination).

**Figure 1:**
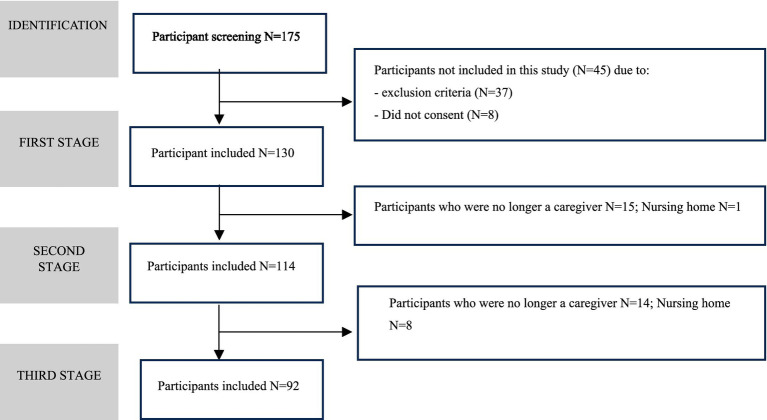
Participant flow diagram.

The study included 130 FCs of persons living with AD at baseline (T1), as illustrated in the flowchart (Figure 1) and detailed in the sample characterization table (Table 1). Participants were predominantly female (78%), included spouses (45%) and daughters (43%), had an average age of 66 years, and resided in rural areas. Baseline assessments included sociodemographic and clinical data, distress, burden, quality of life, health behaviors, forgiveness, caregiving competence, personal gains, family stress, and morning HRV measures to control for time of day (Figure 1).

**Table 1:** Demographic characteristics of persons living with AD and their FCs.

Family caregivers				
	Min	Max	Mean	*SD*
Age	42	92	66.15	13.64
Education	0	20	4.53	3.58
Duration of care (years)	0.5	9	3.98	2.20
	Frequency			%
Sex				
Male	29			22.3
Female	101			77.7
Marital status				
Married	109			83.8
Unmarried	21			16.2
Professional status				
Employed	25			19.2
Unemployed	35			26.9
Retired	70			53.8
Degree of kinship				
Parents	55			42.3
Companion	58			44.6
Others	17			13.1
Caregiving hours				
0–12 h	7			5.4
13–24 h	123			94.6
1st time caregiver				
Yes	92			70.8
No	38			29.2
Chose to be the primary caregiver				
Yes	103			79.2
No	27			20.8
Secondary caregiver				
Yes	71			54.6
No	59			45.4

Person living with Alzheimer disease				
Continuous variables	Min	Max	Mean	*SD*
Age	60	101	85.19	5.97
Education	0	9	1.38	1.91
	Frequency			%
Sex				
Male	44			33.8
Female	86			66.2
Marital status				
Married	66			50.8
Unmarried	64			49.2
	Min	Max	Mean	*SD*
Duration of memory problems	6 months	10 years	3.93	2.29
	Frequency			%
Previous treatments for memory problems				
Yes	44			33.8
No	86			66.2
Stages dementia (CDR)				
Mild	43			33.1
Moderate	37			28.5
Severe	50			38.5

All participants provided written informed consent in accordance with the Declaration of Helsinki. The study was approved by the Ethics Committee for Research in Life and Health Sciences, University of Minho (Ref. CEICSH 46/2020).

### 2.2 Measures

Sociodemographic and clinical questionnaire assessed sociodemographic variables (age, sex, education, professional status, marital status, relationship with the person living with AD, duration of care, caregiving hours); clinical variables of caregivers (support received, presence of illness), and persons living with AD (substance use, memory problems, neurological/psychaitric conditions).

Short-Form Health Survey – SF-36 – (Ware et al., 1993; Severo et al., 2007): The SF-36 questionnaire consists of 36 items across eight subscales of QoL (alphas between 0.80 and 0.93), including physical function, bodily pain, general health, emotional performance, social function, vitality, and mental health. Scores are transformed to a scale of 0 to 100, with higher scores indicating better QoL. Severo et al. (2007) evaluated the Portuguese version’s psychometric properties in 1326 adults, reporting Cronbach’s alpha coefficients of 0.82 for the physical dimension and 0.87 for the mental dimension. In our study, Cronbach’s *α* was 0.92 and McDonald’s *ω* was 0.87 for the physical QoL dimension and for the mental QoL dimension were: Cronbach’s α = 0.87 and McDonald’s *ω* = 0.76.

Zarit Burden Interview Scale – ZBI – (Zarit and Zarit, 1983; Sequeira, 2010): This scale assesses caregivers’ perceived burden, covering various aspects such as health, social, personal, financial, and emotional situations, as well as the type of relationship. The Portuguese version, developed by Sequeira (2010), consists of 22 items. Scores indicate the level of burden, with higher scores reflecting greater burden. The alpha for the Portuguese version is 0.93, while in this study, Cronbach’s alpha and McDonald’s *ω* were 0.94.

Caregiving Competence Scale (Pearlin et al., 1990; GISEF, 2023a): This scale evaluates caregivers’ perceived effectiveness in caregiving tasks, commonly used in FC studies. Scores range from 4 to 16, with higher scores indicating greater caregiving competence. The instrument demonstrated good internal consistency (Cronbach’s *α* = 0.74) (Pearlin et al., 1990), while this study reported a Cronbach’s alpha and McDonald’s *ω* of 0.93.

Personal Gain in Caregiving Scale (Pearlin et al., 1990; GISEF, 2023b): This scale measures personal growth experienced during caregiving. This instrument has strong internal consistency (Cronbach’s *α* = 0.76), used in studies with informal caregivers of persons living with AD. Scores range from 4 to 16, with higher scores indicating greater personal gains. In the present study, the Cronbach’s alpha and the McDonald’s *ω* were 0.92.

Index of Family Relations – IFR – (Hudson, 1993; Pereira and Roncon, 2010): This instrument evaluates personal and social functioning problems within the family, including cohesion, adaptability, conflict, communication, and marital satisfaction. It measures intra-family stress, with higher scores indicating greater stress. The scale comprises 25 items and demonstrated good reliability, with Cronbach’s alphas of 0.95 in the original and Portuguese versions and a McDonald’s *ω* of 0.90 in the present study.

Health Behaviors Questionnaire for Informal Caregivers of Alzheimer’s Patients – HB – (GISEF, 2023c). This questionnaire, adapted from the Lifestyle Questionnaire (Pereira and Pedras, 2009), was validated for this population. It consists of nine items assessing protective and predictive health behaviors on a four-point Likert scale. Higher scores indicate healthier lifestyles. In the present study, the alpha for the nine items was 0.71, and McDonald’s ω was 0.69.

Heartland Forgiveness Scale – HFS – (Thompson et al., 2005; Ikedo et al., 2020): The Heartland Forgiveness Scale (HFS) is an 18-item self-report questionnaire designed to assess the general tendency to forgive (i.e., dispositional forgiveness) others, oneself, and situations. Higher scores indicate greater propensity to forgive. The original and Portuguese versions showed a Cronbach’s alpha of 0.86 for the total scale. In this study, the Cronbach’s alpha was 0.85, and the McDonald’s ω was 0.87.

Depression Anxiety and Stress Scale – DASS-21 – (Lovibond and Lovibond, 1995; Antunes and Mónico, 2015): The DASS-21 is the 21-item short version of a self-report instrument derived from 42 items that assess depression, stress, and anxiety. High scores indicate greater distress. Participants were instructed to answer the questionnaire concerning their role as a family caregiver. The DASS-21 showed a Cronbach’s alpha of 0.93 for the total scale (Henry and Crawford, 2005) and 0.94 in the Portuguese version. The Cronbach’s alpha and the McDonald’s ω, in this study, were 0.97.

Heart Rate Variability – HRV – (Elite HRV, 2019): Stress levels were objectively measured using the CorSense device, known for its accuracy comparable to that of a 5-lead ECG/EKG (Elite HRV, 2019). Heart rate variability (HRV) reflects autonomic nervous system activity and overall well-being, with higher variability indicating better adaptability. The sessions were recorded through the CorSense mobile application and were 5 min long (Shaffer and Ginsberg, 2017) to ensure consistent results. RMSSD, an indicator of parasympathetic activity, is often used to assess cardiovascular health and stress levels. Higher RMSSD values indicate better autonomic nervous system functioning and greater well-being (Nabasny et al., 2022).

### 2.3 Data analysis

A sensitivity analysis was conducted via Monte Carlo simulation (with 1,000 iterations) in R for mixed-effects models, following recommendations in the literature (e.g., Brysbaert and Stevens, 2018). Given the sample size (N = 130) and the variance structure of the fitted model, the analysis estimated the minimum detectable time effect at 80% power (*α* = 0.05, two-sided), corresponding to an approximate change of 1.65 units.

To investigate potential differences between participants who dropped out of the study and those who remained, a binary logistic regression analysis was performed. The dependent variable was dropout status at any assessment point (1 = remained in the study; 0 = dropped out), while the independent variables included FC’s characteristics. The model’s overall fit was assessed using the Omnibus Tests of Model Coefficients, and its performance was evaluated through metrics such as Nagelkerke R^2^ and classification accuracy. The results indicated that the model was not statistically significant (χ^2^(12) = 19.781, *p* = 0.071). The model explained 20.1% of the variance (Nagelkerke R^2^ = 0.201), and the model’s overall classification accuracy was 76.15%, suggesting that FC’s sociodemographic characteristics were not significantly associated with dropout.

A second binary logistic regression was conducted to examine potential differences based on the characteristics of persons living with AD. The dependent variable remained dropout status, while the independent variables included gender, age, marital status, years of education, onset of memory problems, and prior treatments for memory issues. Like the first model, the results showed no statistical significance (χ^2^ (6) = 6.31, *p* = 0.389), with none of the predictor variables reaching significance (*p* > 0.05). The model explained 6.7% of the variance (Nagelkerke R^2^ = 0.067), and the model’s overall classification accuracy was 70.0%, indicating that the persons living with AD’s sociodemographic and clinical characteristics were not predictive of dropout.

Descriptive statistics were utilized to characterize the sample and the mean values of the variables. Pearson correlations were computed, and Cronbach’s alpha was calculated for all scales and subscales. Linear mixed models, suitable for repeated measurements within subjects, were employed to assess changes over time, addressing the correlation between measurements on the same individual. The model was tested using maximum likelihood estimation to determine the point estimates for the regression coefficients, with standard errors based on conventional asymptotic theory (Bates et al., 2015). The primary objective of this study was to examine the trajectories of change and intra-individual variability across the three assessment points. Linear Mixed Models (LMMs) were chosen because they are the ‘gold standard’ for longitudinal data, allowing for the nested structure of the data, modeling random intercepts and slopes, and handling missing data points (Missing at Random) without excluding participants. Mediation analyses were conducted in R, and the mediation package (Tingley et al., 2014) employed bootstrapping with 5.000 subsamples to estimate 95% confidence intervals for indirect effects which were considered significant if the 95% confidence intervals did not include zero (Hayes and Preacher, 2013).

All statistical analyses were performed using the R statistical computing environment (R Development Core Team, 2021). Analyses were controlled for age and clinical dementia rating. Statistical significance was defined as *p*-values below 0.05.

## 3 Results

### 3.1 Differences over time

**Figure 2:**
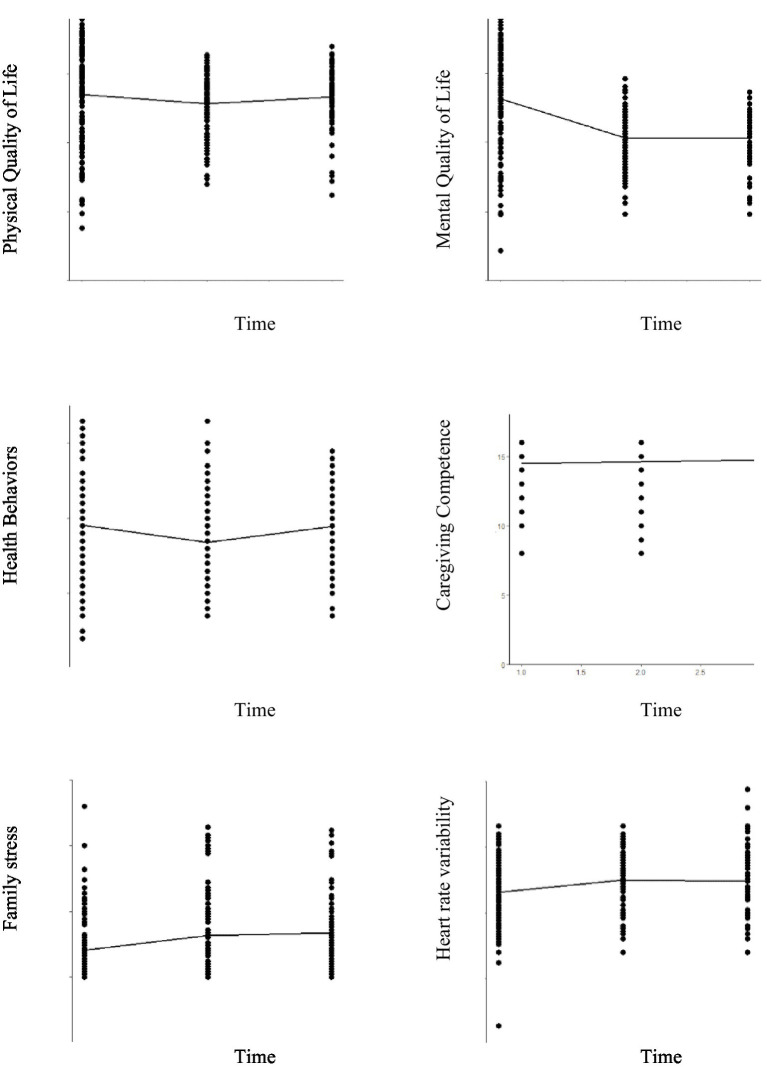
Graphical representation of differences over time.

Physical QoL and health behaviors significantly decreased from T1 to T2 (*β* = −3.408, *p* < 0.001; *β* = −2.324, *p* < 0.001) and significantly increased from T2 to T3 (*β* = 2.037, *p* = 0.031; *β* = 2.155, *p* < 0.001) (Table 2; Figure 2). Mental QoL significantly decreased from T1 to T2 (*β* = −14.145, *p* < 0.001) and from T1 to T3 (*β* = −14.717, *p* < 0.001) (Table 2; Figure 2).

**Table 2:** Differences over time.

Response variable	Physical quality of life	Mental quality of life	Burden	Heart rate variability	Caregiving competence	Personal gains	Health behaviors	Distress	Forgiveness	Family stress
Fixed effects	Beta (SE)	Beta (SE)	Beta (SE)	Beta (SE)	Beta (SE)	Beta (SE)	Beta (SE)	Beta (SE)	Beta (SE)	Beta (SE)
Intercept	94.339 (5.215)***	69.931 (5.329)***	49.840 (6.722) ***	45.540 (5.588)***	17.253 (0.818)***	17.272 (0.863)***	18.115 (1.943)***	12.810 (5.735)*	104.148 (6.068)***	37.429 (5.262)***
Time 2	−3.408 (1.233)**	−14.145 (1.435)***	3.005 (1.683)	4.295 (1.467)**	0.148 (0.040)***	0.026 (0.020)	−2.324 (0.538)***	1.986 (1.647)	0.465 (1.841)	5.558 (1.634) ***
Time 3	−1.371 (1.423)	−14.717 (1.670)***	3.460 (1.998)	4.155 (2.182)*	0.233 (0.054)***	0.129 (0.072)	−0.169 (0.621)	0.415 (1.871)	−1.689 (1.978)	6.693 (1.775)***
Age	−0.421 (0.074)***	−0.086 (0.076)	−0.023 (0.095)	0.180 (0.079)*	−0.045 (0.012)***	−0.045 (0.012)***	0.021 (0.028)	0.036 (0.081)	−0.127 (0.086)	−0.023 (0.075)
CDR 2	1.180 (2.572)	−0.580 (2.623)	11.384 (3.093)**	2.472 (2.735)	0.116 (0.405)	0.029 (0.428)	−0.751 (0.955)	3.641 (2.820)	−9.234 (2.979)**	0.773 (2.584)
CDR 3	2.397 (2.399)	3.988 (2.451)	−0.614 (3.093)	−0.716 (2.576)	0.406 (0.012)	0.301 (0.397)	−0.397 (0.894)	−4.047 (2.636)	4.386 (2.790)	−2.243 (2.417)

*p < 0.05; **p < 0.01; ***p < 0.001. CDR – Clinical Dementia Rating.

HRV and family stress significantly increased from T1 to T2 (*β* = 4.295, *p* = 0.004; *β* = 5.558, *p* < 0.001) and from T1 to T3 (*β* = 4.155, *p* = 0.038; *β* = 6.693, *p* < 0.001) (Table 2; Figure 2).

Caregiving competence significantly increased from T1 to T2 (*β* = 0.148, *p* < 0.001), and from T1 to T3 (*β* = 0.233, *p* < 0.001) and from T2 to T3 (*β* = 0.085, *p* = 0.034) (Table 2; Figure 2).

No significant differences were found in burden, personal gains, distress, and forgiveness (Table 2).

### 3.2 Mediation analysis

Mediation analysis was also used to examine whether forgiveness mediated the relationships between distress and burden and between distress and QoL. The indirect effect was significant, between distress and burden *β* = 0.1156, *p* < 0.001, 95% CI [0.0683; 0.1700] and between distress and physical QoL *β* = 0.0499, *p* = 0.0044, 95% CI [−0.0877; −0.0200]. Forgiveness did not mediate the relationship between distress and mental QoL (indirect effect: *β* = −0.0185, *p* = 0.33, 95% CI [−0.0559; 0.020]), (Table 3).

Health behaviors did not mediate the relationship between HRV and burden (indirect effect: *β* = −0.00231, *p* = 0.80, 95% CI [−0.02244; 0.020]) (Table 3).

**Table 3:** Mediation effects.

Independent variable	Mediator		Dependent variable
	Estimate	*p*-value	95% CI
Heart rate variability → Health behaviors → Burden			
Indirect effect	−0.00231	0.80	(−0.02344; 0.0200)
Direct effect	0.12634	0.022	(0.01836; 0.2400)
Total effect	0.12401	0.022	(0.01775; 0.2300)
Distress → Forgiveness → Mental quality of life			
Indirect effect	−0.0185	0.33	(−0.0559; 0.0200)
Direct effect	−0.4828	<0.001	(−0.5673; −0.4000)
Total effect	−0.5012	<0.001	(−0.5780; −0.4200)
Distress → Forgiveness → Physical quality of life			
Indirect effect	−0.0499	0.0044	(−0.0877; −0.0200)
Direct effect	−0.293	<0.001	(−0.3732; −0.2100)
Total effect	−0.3427	<0.001	(−0.4171; −0.2700)
Distress → Forgiveness → Burden			
Indirect effect	0.1156	<0.001	(0.0683; 0.1700)
Direct effect	0.5633	<0.001	(0.4621; 0.6700)
Total effect	0.6792	<0.001	(0.58; 0.7700)

## 4 Discussion

The study aimed to analyze longitudinal changes in psychosocial and physiological factors among FCs of persons living with AD, controlling for age and clinical dementia rating. QoL scores, particularly in physical and mental dimensions, displayed notable patterns. Physical QoL initially decreased from T1 to T2, likely due to challenges like infection concerns and disrupted routines during COVID-19, as suggested by previous research (Cohen et al., 2021). However, QoL improved from T2 to T3, indicating potential adaptive responses or support mechanisms (Contreras et al., 2021; Ercoli et al., 2021; Jeste et al., 2021). Conversely, mental QoL consistently declined across T1, T2, and T3, highlighting persistent psychological strain among caregivers and underscoring the urgent need for targeted interventions (Damian et al., 2023). The sustained decline underscores the chronic impact of caregiving responsibilities, compounded by various stressors such as emotional distress and social isolation (Cohen et al., 2021; Savla et al., 2021). The continuous decrease suggests a systemic issue that requires ongoing, focused support initiatives to adequately address caregivers’ mental well-being. In other words, FCs may adapt emotionally to persistent stressors without necessarily perceiving their overall mental quality of life as preserved. Additionally, the longitudinal decline in mental QoL may reflect cumulative caregiving strain. Chronic exposure to high caregiving demands can lead to gradual erosion of perceived vitality, social engagement, and sense of personal fulfillment, even in the absence of escalating acute emotional distress. This pattern is consistent with stress process perspectives, suggesting that prolonged exposure to stressors may produce enduring impacts on global well-being through mechanisms of psychological wear-and-tear and reduced recovery opportunities.

Despite evolving pandemic circumstances, no significant changes in FCs’ burden were observed, suggesting a stable perception of burden. This consistency may indicate resilience or effective coping strategies among caregivers, in contrast to previous findings of increasing burden over time (Connors et al., 2019; Kawaharada et al., 2019; Reed et al., 2020). However, Savla et al. (2021) found decreased burden among FCs with fewer COVID-19 concerns and increased support from family and friends. Similarly, our results align with findings showing consistent burden levels among FCs with access to services or in residential care, contrasting with increased burden among those without services.

HRV increased notably from T1 to T2 and T3, suggesting a positive physiological response or adaptation in FCs, possibly indicating enhanced coping mechanisms or improved overall well-being. This trend aligns with the sustained increase in caregiving competence, indicating growing confidence and proficiency in caregivers’ roles over time, despite various stressors, including COVID-19 (Kim et al., 2018).

No significant changes in personal gains were observed, indicating a stable self-perception among FCs. This aligns with findings from other studies suggesting that FCs may respond to stress by seeking social support or professional help (Chunga et al., 2021; Quinn et al., 2022). Quinn et al. (2022) studied the impact of the pandemic on FCs in England and Wales, finding that while many felt their healthcare needs were negatively affected, a significant proportion coped well, adapting communication methods and support networks. Similarly, Hanna et al. (2022) identified resilience factors in both persons living with AD and FCs during COVID-19, highlighting the importance of protective resources and individual coping mechanisms in managing caregiving challenges.

Health behaviors initially decreased from T1 to T2 but rebounded from T2 to T3. This fluctuation suggests that FCs experienced disruptions to their routines but made efforts to reinstate healthier habits (Bailey et al., 2022; Cohen et al., 2021).

Distress levels remained stable over time, suggesting consistent emotional well-being among FCS, even when considering age and clinical dementia rating (Terlizzi and Villarroel, 2020; Villarroel and Terlizzi, 2020). The resilience towards distress may indicate effective coping strategies or adequate support received by FCs, possibly through increased social support or accessing mental health resources (Wiegelmann et al., 2021). These findings align with those of Damian et al. (2023), who found stable distress levels but declining QoL and health behaviors among caregivers during the pandemic. Further research could explore specific coping mechanisms or interventions contributing to this stability in distress levels.

No significant changes in forgiveness over time were observed among FCs, suggesting that caregivers may have stable coping mechanisms, as noted by DeCaporale-Ryan et al. (2016), or that forgiveness may be an enduring aspect of their disposition, as suggested by McGee et al. (2021).

Family stress increased from T1 to T2 and T3, indicating potential challenges for FCs. The progression of caregiving responsibilities, as the health of the person living with AD declines, may contribute to this rise (Reed et al., 2020). Additionally, external factors, such as the impact of a pandemic, could exacerbate family stress over time (Bailey et al., 2022; Cohen et al., 2021). Stressors in the socio-cultural, economic, or health domains can affect the family environment, heightening caregiver stress. Taken together, these findings are consistent with evidence that FCs’ coping efforts may shift over time in response to accumulating stressors (Savla et al., 2021). Despite increased caregiving duties and limited access to formal support (Roach et al., 2021), many caregivers identified someone who could furnish support if required (Hanna et al., 2022). The result implies that FCs had more family contact and support during the pandemic, coinciding with the initiation of some remote home support services. However, many FCs stopped receiving assistance due to COVID-19 risks, leading to increased caregiving duties (Giebel et al., 2020). Their study also highlighted challenges arising from reduced perceived social support, such as a sense of loss of control and the need to adjust to the ‘new normal’ (Giebel et al., 2020).

The results showed that forgiveness mediated the relationship between distress and burden, as well as distress and physical QoL, suggesting that forgiveness could buffer the impact of distress on caregivers. Although forgiveness remained stable over time, it consistently mitigated the negative effects of distress. This highlights forgiveness as a resilient coping mechanism for FCs, offering potential avenues for interventions to enhance caregiver well-being. These findings align with existing literature on the positive effects of forgiveness in caregiving contexts (Levy et al., 2021; McGee et al., 2021).

Forgiveness has been found to be an effective coping strategy, reducing stress and promoting positive adaptations (Strelan, 2019). Research indicates a positive association between forgiveness and QoL, with therapeutic benefits in alleviating psychological distress (Kaleta and Mróz, 2020). Additionally, forgiveness may mediate the relationship between depression symptoms and distress in FCs (Rasmussen et al., 2019). By mitigating stress and resolving negative emotions, forgiveness can foster better outcomes in caregiving contexts (Kaleta and Mróz, 2020).

The findings of the present study challenged our initial expectations regarding the mediation role of forgiveness in the relationship between distress and mental QoL. This unexpected outcome prompts further exploration into the intricate dynamics at play. It suggests that forgiveness may not fully account for the association between distress and mental QoL, hinting at the involvement of other unexplored variables or complex interactions. Moreover, factors like age, education level, and the intensity of caregiving responsibilities may influence caregivers’ coping mechanisms and their ability to engage in forgiveness, potentially affecting forgiveness’s mediating role. The emotional dynamics within spousal or parental relationships (44.6 and 42.3% respectively) may influence the forgiveness process differently. With persons living with AD averaging 85 years old, potentially evincing advanced age and more severe cognitive impairment, additional research exploring other populations could shed light on why forgiveness may not mediate the distress-mental QoL relationship in this context. This finding is in disagreement with previous research suggesting that forgiveness has positive health effects when used as a coping mechanism to reduce stress associated with anger and revenge. Forgiveness has been associated with health-enhancing factors like hope and optimism while inhibiting health-eroding factors such as self-blame and pessimism (Davis et al., 2015). Forgiveness has also been linked to lower psychological distress, improved life satisfaction and health, and better endocrine and immunological profiles (Toussaint et al., 2015). Additionally, compassion has been correlated with psychological well-being indicators like forgiveness, positive thinking, positive affect, and empathy (Wu et al., 2019). Caregiver forgiveness studies suggest that reducing FCs’ burden perception might involve forgiving the behavioral challenges of the person living with AD.

Health behaviors did not mediate the relationship between HRV and burden, possibly due to sociodemographic differences between FCs and persons living with AD. With an average age of 66 years old and lower levels of education, FCs may have lower health literacy and engage less in healthful behaviors, potentially affecting their health outcomes (Dao and Fu, 2023). The findings align with previous research indicating that higher literacy levels are linked to better health literacy among caregivers of disabled older adults (Deshpande et al., 2023). The average duration of care was approximately four years, suggesting that long-term caregiving may lead to adaptive behaviors, reducing the impact of HRV on burden. Given that most caregivers dedicate over 24 h daily and more than 70% are first-time caregivers, their focus on caregiving duties may overshadow the influence of personal health behaviors on burden. This complex caregiving environment underscores the need for comprehensive support systems to address caregivers’ physical and mental health challenges, underscoring the critical link between caregivers’ well-being and the quality of care they provide (Rawat et al., 2023; Sehar et al., 2022).

## 5 Limitations

While this longitudinal study provides valuable insights into the experiences of FCs of persons living with AD during the COVID-19 pandemic, some limitations should be considered when interpreting the findings. Most psychosocial variables were assessed using self-report measures, which may introduce common-method bias and inflate associations due to shared measurement variance or social desirability. Future research should incorporate additional objective or multi-informant data where feasible. The sample consisted exclusively of Portuguese family caregivers from Northern Portugal, which may limit generalizability, as caregiving norms, access to support, and coping processes, such as forgiveness, are culturally embedded. Moreover, although data were collected during the COVID-19 pandemic, individual-level pandemic exposures (e.g., infection, bereavement, financial strain) were not systematically assessed and may have influenced outcomes. Finally, the study focused solely on caregivers and did not include dyadic data from persons living with AD, precluding a more relational understanding of reciprocal influences within the caregiving context.

The present study provides preliminary longitudinal evidence for the mediating role of forgiveness in the context of caregiver distress and burden/physical QoL within a specific cultural and pandemic context. However, these findings require replication in more diverse samples using multi-method approaches. Future research should aim to recruit larger, more diverse samples to build upon the findings by employing longitudinal mediation analysis to further explore the specific causal pathways between the psychological variables, HRV, and burden/QoL, across time. Also, the use of dyadic designs, objective, and ecological assessments will build a more robust understanding of the caregiving stress process.

## 6 Conclusion

The results highlight the need for targeted support interventions. Future research could further investigate the factors behind increased family stress and delve into the intricate dynamics within caregiving families. Understanding these dynamics is vital for improving support services and enhancing the well-being of caregivers and their families. According to results, effective programs should focus on maintaining caregivers’ well-being, enhancing their QoL, and boosting their confidence and competence in caregiving roles. Healthcare professionals and support services should acknowledge the complex relationship between family stress and caregiver distress. Targeted interventions such as family counseling, respite care, and educational programs can better equip families with effective coping strategies, ultimately supporting caregivers in providing better care.

Encouraging forgiveness-based interventions could reduce the burden among distressed caregivers and enhance their overall well-being. Collaborative efforts between public health departments, care workers, and families are crucial for ensuring continuity of care, especially during unique COVID-19 circumstances. Future research should integrate objective measures, such as biomarkers or physiological parameters, alongside subjective assessments, could offer a comprehensive understanding of the physical and psychological impact of caregiving on QoL.

## Data Availability

The raw data supporting the conclusions of this article will be made available by the authors, without undue reservation.
